# Two Novel Flavin-Containing Monooxygenases Involved in Biosynthesis of Aliphatic Glucosinolates

**DOI:** 10.3389/fpls.2016.01292

**Published:** 2016-08-29

**Authors:** Wenwen Kong, Jing Li, Qingyue Yu, Wei Cang, Rui Xu, Yang Wang, Wei Ji

**Affiliations:** ^1^Department of Plant Biotechnology, College of Life Science, Northeast Agricultural UniversityHarbin, China; ^2^Department of Cell Biology, College of Life Science, Northeast Forestry UniversityHarbin, China

**Keywords:** *Arabidopsis thaliana*, flavin-containing monooxygenase (FMO), glucosinolates, *S*-oxygenation, expression pattern

## Abstract

Glucosinolates, a class of secondary metabolites from cruciferous plants, are derived from amino acids and have diverse biological activities, such as in biotic defense, depending on their side chain modification. The first structural modification step in the synthesis of aliphatic (methionine-derived) glucosinolates—*S*-oxygenation of methylthioalkyl glucosinolates to methylsulfinylalkyl glucosinolates—was found to be catalyzed by five flavin-containing monooxygenases (FMOs), FMO_GS-OX1-5_. Here, we report two additional FMO_GS-OX_ enzymes, FMO_GS-OX6_, and FMO_GS-OX7_, encoded by *At1g12130* and *At1g12160*, respectively. The overexpression of both *FMO_GS-OX6_* and *FMO_GS-OX7_* decreased the ratio of methylthioalkyl glucosinolates to the sum of methylthioalkyl and methylsulfinylalkyl glucosinolates, suggesting that the introduction of the two genes converted methylthioalkyl glucosinolates into methylsulfinylalkyl glucosinolates. Analysis of expression pattern revealed that the spatial expression of the two genes is quite similar and partially overlapped with the other *FMO_GS-OX_* genes, which are primarily expressed in vascular tissue. We further analyzed the responsive expression pattern of all the seven *FMO_GS-OX_* genes to exogenous treatment with abscisic acid, 1-aminocyclopropane-1-carboxylic acid (ACC), jasmonic acid (JA), salicylic acid, indole-3-acetic acid (IAA), and low and high temperatures. Although these genes showed same tendency toward the changing stimulus, the sensitivity of each gene was quite different. The variety in spatial expression among the *FMO_GS-OX_* genes while responding to environmental stimulus indicated a complex and finely tuned regulation of glucosinolates modifications. Identification of these two novel FMO_GS-OX_ enzymes will enhance the understanding of glucosinolates modifications and the importance of evolution of these duplicated genes.

## Introduction

Glucosinolates (GSLs) are amino acid-derived natural products primarily present in plants belonging to the Brassicaceae family, such as cabbage, broccoli, and the model plant *Arabidopsis thaliana*. Upon wounding or mastication, GSLs are hydrolyzed by myrosinases (thioglucosidases) and release hydrolysis products, primarily isothiocyanates and nitriles (Halkier and Gershenzon, 2006; Bones and Rossiter, 2006; Zhang et al., 2006). These breakdown products exert diverse biological effects such as induction of direct toxic effects or other defense responses against pathogens and generalist herbivores (Bednarek et al., 2009; Clay et al., 2009; Hopkins et al., 2009; Laluk et al., 2012). In humans, the isothiocyanates derived from some aliphatic (methionine-derived) GSLs are considered to have numerous health benefits including potent anti-cancer property (Fahey et al., 1997, 2002; Rose et al., 2000; Zalcmann and Talalay, 2001). The most well-studied isothiocyanate is sulforaphane, which is derived from 4-methylsulfinylbutyl GSL; it can decrease the risk of various cancers such as breast cancer, prostate cancer, gastric cancer, and skin cancer (Fahey et al., 2002; Rose et al., 2005; Talalay et al., 2007; Atwell et al., 2015; Chang et al., 2015; Fofaria et al., 2015). Aliphatic GSLs are synthesized in three steps—elongation of methionine chain, formation of GSL core structure, and modification of side chain. The diverse biological activities of GSLs are largely dependent on the chemical modifications in their side chain.

The basic aliphatic glucosinolate molecules, methylthioalkyl (MT) GSLs, which contain only the core structure, may undergo a variety of secondary modifications such as oxidation, hydroxylation, or desaturation. The first step in side-chain modification is *S*-oxygenation, which converts MT GSLs to methylsulfinylalkyl (MS) GSLs. Thus, *S*-oxygenation is of biological as well as biochemical interest because it influences not only the biosynthesis of MS GSLs, but also the further modification and the resulting activity of their hydrolysis products.

**FIGURE 1:**
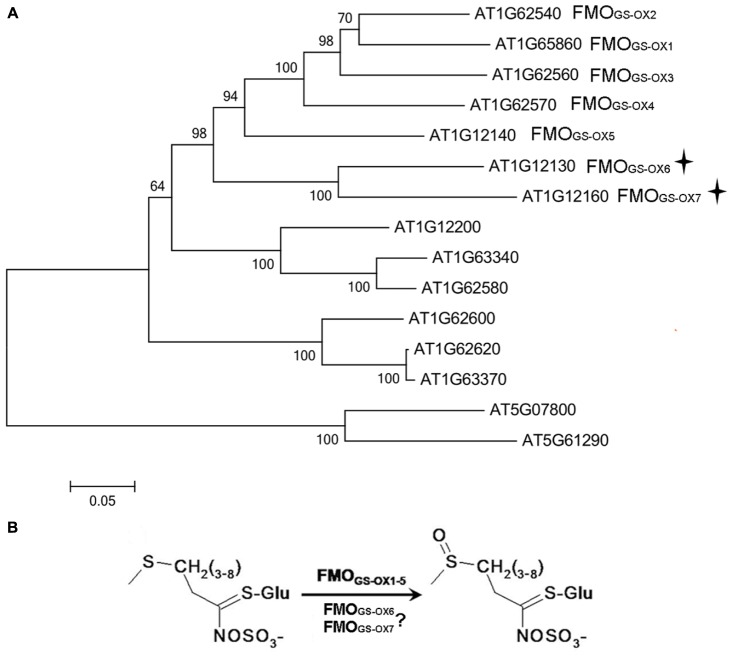
**(A)** Phylogenetic tree of *Arabidopsis* flavin-containing monooxygenases (FMOs) possessing the conserved GS-OX domains. **(B)** FMO_GS-OX6_ and FMO_GS-OX7_ are predicted to catalyze *S*-oxygenation of methylthioalkyl (MT) Glucosinolates (GSLs) to methylsulfinylalkyl (MS) GSLs.

Five flavin-containing monooxygenases (FMOs), FMO_GS-OX1-5_, have been identified to catalyze *S*-oxygenation during the conversion of MT GSLs to MS GSLs (Hansen et al., 2007; Li et al., 2008). In phylogenetic analysis of FMOs containing the conserved FMO_GS-OX_ domains in *Arabidopsis*, At1g12130 and At1g12160 clustered to the same subclade and, together with FMO_GS-OX1-5_, formed a seven-protein group (**Figure 1A**). In a previous study, a phylogenetic tree of plant-derived FMOs in rice, *Arabidopsis*, and poplar was analyzed, and this seven-protein group was involved in the predicted *S*-oxygenation clade and was present only in the GSL-producing *Arabidopsis* plant. Thus, these genes were predicted to be involved in the *S*-oxygenation of GSL (**Figure 1B**). Further, another FMO-coding gene, *At1g12200*, was suggested as a candidate *FMO_GS-OX_* due to its strong co-expression with other GSL biosynthetic genes in response to *Sclerotinia sclerotiorum* (Stotz et al., 2011) infection. Therefore, in our study, we investigated *At1g12130, At1g12160*, and *At1g12200* as FMO_GS-OX_ candidate genes. It was discovered that both At1g12130 (named FMO_GS-OX6_) and At1g12160 (named FMO_GS-OX7_) are involved in *S*-oxygenation of aliphatic GSLs, while At1g12200 does not possess the expected activity.

To better understand the coordination of these *FMO_GS-OX_*s and the upstream aliphatic GSL biosynthetic genes, responsive expression pattern of these genes toward several hormone treatments and environmental conditions are detected. Integrating the knowledge of catalysis, distribution and responsive pattern of these FMO_GS-OX_s, we speculate that on one hand, these duplicated genes are redundant and might functionally compensate and increase genetic robustness, whereas on the other hand, they show delicate functional variation and possibly contribute to the complex and finely tuned regulation of GSLs modification.

## Materials and Methods

### Generation of Phylogenetic Tree

All the FMO protein sequences with conserved GS-OX domains in *Arabidopsis* were obtained from NCBI (Marchler-Bauer et al., 2015) and the full-length amino acid sequences of these proteins were aligned using ClustalX with default parameters. A phylogenetic tree was constructed using the neighbor-joining method with Mega software (version 5.0). Bootstrap support for the topology was estimated from 1000 replicates, and nodes occurring in less than 50% of the replicates were collapsed.

### Plant Material and Growth Conditions

Wild-type (Col-0) and mutants of *Arabidopsis* seeds were obtained from *Arabidopsis* Biological Resource Center. Genotype of *FMO_GS-OX6_* T-DNA insertion mutant (*SALK_209008*) was analyzed by PCR using the following three primers, 5′-CCGCCTAATTCAATCAGCTC-3′, 5′-GAGGCTCAGTGAGATGACC-3′, and the T-DNA-specific primer LBa1 5′-TGGTTCACGTAGTGGGCCATCG-3′. Genotype of *FMO_GS-OX7_* T-DNA insertion mutant (*SALK_062129*) was analyzed by PCR using the following three primers, 5′-AAACCGTGAAGATTGCAATTG-3′, 5′-GACGAGGCTCAGTGAAGTGAC-3′, and the T-DNA-specific primer LBa1 5′-TGGTTCACGTAGTGGGCCATCG-3′. Genotype of *At1g12200* T-DNA insertion mutant (*SALK_136282*) was analyzed by PCR using the following three primers, 5′-CCGCCTAATTCAATCAGCTC-3′, 5′-GAGGCTCAGTGAAGTGACC-3′, and the T-DNA-specific primer LBa1 5′-TGGTTCACGTAGTGGGCCATCG-3′.

Plants were grown in a growth chamber with 16 h light/8 h dark photoperiod at a photosynthetic photon flux density of 100 μmolm^-2^ s^-1^ at 20 and 70% relative humidity, respectively.

### Plasmid Construction and Plant Transformation

To prepare the overexpression constructs *35S::FMO_GS-OX6_*, 3*5S::FMO_GS-OX7_*, and 3*5S::At1g12200*, coding sequences (CDS) of *FMO_GS-OX6_, FMO_GS-OX7_* and *At1g12200* were amplified through reverse transcription-PCR (RT-PCR) method. The total RNA from plant was isolated with TRIzol reagent according to the manufacturer’s instructions. First-strand cDNA was synthesized using the SupermoIII RT kit (Bioteke). The CDS of *FMO_GS-OX6_, FMO_GS-OX7_* and *At1g12200* were amplified with Pfu Turbo Cx Hotstart DNA polymerase (Stratagene) from the first-strand products using the following primers, respectively: 5′-GGCTTAAUATGACACCACCGCCTAATTC-3′, 5′-GGTTTAAUTTATGTAGACCAAACTTTGCTCG-3′ and 5′-GGCTTAAUATGACCAGCGTAATCACCTC-3′, 5′-GGTTTAAUCTAAGAAGAACAATCTTGGTTCG-3′, and 5′-ATGGCAACGAGTCATCCTGA-3′, 5′-TTAGGTTTTGAGCATCGGCAAAAG-3′. The PCR products were then cloned into the expression vector pCAMBIA230035Su using the USER Cloning method as described by Nour-Eldin et al. (2006).

To identify the spatial expression pattern of *FMO_GS-OX6_* and *FMO_GS-OX7_*, two constructs *FMO_GS-OX6_Pro::GUS* (*GUS* was driven by the promoter of *FMO_GS-OX6_*) and *FMO_GS-OX7_Pro::GUS* (*GUS* was driven by the promoter of *FMO_GS-OX7_*), respectively, were prepared. For *FMO_GS-OX6_Pro::GUS*, a 999bp DNA fragment containing the *FMO_GS-OX6_* promoter was amplified with Pfu Turbo Cx Hotstart DNA polymerase (Stratagene) from genomic DNA using the PCR primers 5′-GGCTTAAUGAGTGGTTAATGTGCAACATCAGC-3′ and 5′-GGTTTAAUAGTATCAGTCAAAGTATTTGTTTCCTCG-3′. For *FMO_GS-OX7_Pro::GUS*, a 417 bp DNA fragment containing the *FMO_GS-OX7_* promoter was amplified with Pfu Turbo Cx Hotstart DNA polymerase (Stratagene) from genomic DNA using the PCR primers 5′-GGCTTAAUAACGGGATTTTTGATTGGTT-3′ and 5′-GGTTTAAUTGTCAAGTCAATTAATGTCAATCTATCAG-3′. The PCR products were then cloned into the expression vector pCAMBIA3300 NLS-GUS by using the USER method as described by Nour-Eldin et al. (2006). pCAMBIA3300 NLS-GUS is generated from the original vector pCAMBIA3300 by adding the NLS (nuclear localization signal) and CDS of the *GUS* with User cloning sites (Nour-Eldin et al., 2006).

Using *Agrobacterium*-mediated transformation, all the constructs were transformed into *Arabidopsis* using the floral-dip method (Clough and Bent, 1998). *T*_0_ transgenic plants were selected on 1/2 Murashige and Skoog (MS) medium containing 50 μg⋅mL^-1^ kanamycin. For each construct, three independent confirmed *T*_2_ transgenic lines were used in the following analysis.

### GSL Extraction and Analysis

*35S::FMO_GS-OX6_, 35S::FMO_GS-OX7_*, wild-type, *FMO_GS-OX6_* knockout mutant, and *FMO_GS-OX7_* knockout mutant plants were grown simultaneously for 24 days. Individual leaves from each plant were harvested for GSL measurement. 50 to 100 mg leaves were used for the extraction. GSL extraction was performed as previously described (Hansen et al., 2007). High performance liquid chromatography (HPLC) analysis was performed by using the method described by Pang et al. (2009).

### Spatial Expression Analysis

The histochemical detection of *GUS* expression was performed as previously described (Jefferson et al., 1987). Plant materials were cut and incubated in substrate solution at 37°C for 12 h followed by removal of chlorophyll by submerging the samples in 96% ethanol. The samples were examined and photographed using a stereo microscope (Nikon SMZ1270) or a research microscope (Olympus CX21).

For analysis of the responsive expression pattern, seeds of *FMO_GS-OX6_Pro::GUS, FMO_GS-OX7_Pro::GUS*, and wild-type plants were vernalized at 4°C for 72 h and allowed to germinate in 1/2 MS medium for 5 days. The plants were then transferred to 1/2 MS medium containing 50 μM methyl jasmonate (MeJA), 200 μM salicylic acid (SA), and 10 μM abscisic acid (ABA), respectively, and were treated for 48 h. The whole seedling was used for GUS detection using the abovementioned method.

### Responsive Gene Expression Analysis

Wild-type seeds were vernalized at 4°C for 72 h and allowed to germinate in 1/2 MS medium for 5 days. The seedlings were then transferred to 1/2 MS medium containing 100 μM indole acetic acid (IAA), 200 μM SA, 50 μM MeJA, 20 mM 1-aminocyclopropane-1-carboxylic acid (ACC), and 10 μM ABA, respectively. For the cold stress and heat stress treatments, seedlings were transferred to 4 and 30°C growing chamber, respectively. All the treatments were performed for 24 h.

Total RNA of the treated and control plants were isolated using TRIzol reagent (Invitrogen) and first-strand cDNA was synthesized using the SupermoIII RT kit (Bioteke). The primer pairs used in qRT-PCR for the detection of genes are listed in Supplementary Table S1. qRT-PCR was performed using SYBR Green Master Mix on an ABI 7500 sequence detection system. Relative transcript levels were normalized using *ACTIN II* as a control.

## Results

### Phylogenetic Analysis of *FMO_GS-OX_* Genes in *Arabidopsis*

Flavin-containing monooxygenases protein sequences containing the conserved FMO_GS-OX_ domains were obtained from TAIR^1^ and were aligned using ClustalX. It was found that At1g12130 and At1g12160 were located in the same subclade, and together with the annotated FMO_GS-OX1-5_ formed a seven-protein group (**Figure 1A**), while At1g12200 was located in a subclade adjacent to the seven-protein group (**Figure 1A**).

Because both the overexpressor and knockout mutant of *At1g12200* did not show the expected phenotype (Supplementary Tables S2 and S3), the possibility of At1g12200 to be a FMO_GS-OX_ could be excluded. Thus, in the following study, we focused on *At1g12130* (named *FMO_GS-OX6_*) and *At1g12160* (named *FMO_GS-OX7_*) (**Figure 1B**).

### Overexpression of *FMO_GS-OX6_* and *FMO_GS-OX7_* Altered MT GSLs and MS GSLs Profile

To confirm whether FMO_GS-OX6_ and FMO_GS-OX7_ possess *S*-oxygenation activity, transgenic plants *35S::FMO_GS-OX6_* and *35S::FMO_GS-OX7_* were generated and three independent lines of each *FMO_GS-OX6_* and *FMO_GS-OX7_* overexpressors were, respectively, used to analysis GSLs profile. Data of the line with the most significant phenotype was shown in **Tables 1** and **2**. These transgenic plants were confirmed to have much higher expression level than that in wild-type plants by RT-PCR analysis (**Supplementary Figure S1**). For each plant, the content of GSLs was detected in leaves and seeds of segregating progeny obtained from a heterozygous transgenic parent, thus the possible maternal effects could be minimized. Since the conversion of MT GSLs to MS GSLs rather than the absolute content of MS GSLs could better represent the *S*-oxygenation activity, MT: (MT+MS) was calculated from the HPLC data. Aliphatic GSLs with side chains of different lengths, including C3 (propyl), C4 (butyl), C5 (pentyl), C6 (hexyl), C7 (heptyl), and C8 (octyl), in the leaf and seed tissues of *35S::FMO_GS-OX6_, 35S::FMO_GS-OX7_*, and wild-type are shown in **Tables 1** and **2**. In the seed, MT:(MT+MS) of all the detectable GSLs except propyl, were significantly lower in both *35S::FMO_GS-OX6_* and *35S::FMO_GS-OX7_* than in the wild-type. In the leaf, MT:(MT+MS) was lower in *35S::FMO_GS-OX7_* than in the wild-type, while it was not significantly changed in *35S::FMO_GS-OX6_.* The significantly decreased MT:(MT+MS) in the seed tissue of the two overexpressors suggested that the FMO_GS-OX6_ and FMO_GS-OX7_ possess *S*-oxygenation activity for both short-chain and long-chain GSLs. The weak phenotype of overexpressors in the leaf tissue had been previously observed for other FMO_GS-OX_ enzymes (Hansen et al., 2007; Li et al., 2008); this is possibly because of the low content of the substrate (MT GSLs) and the high content of the product (MS GSL) in the leaf, which showed impaired MT converting reaction.

**Table 1:** Glucosinolates profile in *35S::FMO_GS-OX6_*.

MT:(MS+MT)	Leaf tissue			Seed tissue		
	WT	*35S:: FMO_GS-OX6_*	*P*-value	WT	*35S:: FMO_GS-OX6_*	*P*-value
Propyl GSL (C3)	0.11 ± 0.009	0.10 ± 0.005	NS	0.02 ± 0.001	0.01 ± 0.000	NS
Butyl GSL (C4)	0.28 ± 0.021	0.27 ± 0.022	NS	0.90 ± 0.010	0.57 ± 0.021	<0.05
Pentyl GSL (C5)	0.33 ± 0.013	0.31 ± 0.011	NS	0.75 ± 0.039	0.41 ± 0.024	<0.05
Hexyl GSL (C6)	ND	ND		ND	ND	
Heptyl GSL (C7)	0.30 ± 0.012	0.31 ± 0.012	NS	0.77 ± 0.025	0.40 ± 0.019	<0.05
Octyl GSL (C8)	0.17 ± 0.011	0.17 ± 0.012	NS	0.31 ± 0.013	0.14 ± 0.011	<0.05

*Data presented are mean values of MT:(MS+MT) ± Standard Error for at least three replicates per sample. *P*-value for MT:(MS+MT) differences between the two genotypes were determined by Student’s *t*-test. ND means given GSL was not detectable; therefore, no statistical analyses were conducted. NS means non-significant *P*-value (*P* > 0.05).*

**Table 2:** Glucosinolates profile in 35S::*FMO_GS-OX7_*.

MT:(MS+MT)	Leaf tissue			Seed tissue		
	WT	*35S:: FMO_GS-OX7_*	*P*-value	WT	*35S:: FMO_GS-OX7_*	*P*-value
Propyl GSL (C3)	0.09 ± 0.004	0.06 ± 0.003	<0.05	0.03 ± 0.007	0.04 ± 0.010	NS
Butyl GSL (C4)	0.39 ± 0.014	0.11 ± 0.003	<0.05	0.83 ± 0.024	0.59 ± 0.011	<0.05
Pentyl GSL (C5)	0.43 ± 0.009	0.30 ± 0.010	<0.05	0.74 ± 0.021	0.38 ± 0.012	<0.05
Hexyl GSL (C6)	ND	ND		ND	ND	
Heptyl GSL (C7)	0.35 ± 0.011	0.20 ± 0.009	<0.05	0.66 ± 0.012	0.31 ± 0.012	<0.05
Octyl GSL (C8)	0.13 ± 0.008	0.06 ± 0.005	<0.05	0.32 ± 0.014	0.13 ± 0.008	<0.05

*Data presented are mean values of MT:(MS+MT) ± Standard Error for at least three replicates per sample. *P*-value for MT:(MS+MT) differences between the two genotypes were determined by Student’s *t-*test. ND means given GSL was not detectable; therefore, no statistical analyses were conducted. NS means non-significant *P*-value (*P* > 0.05).*

T-DNA knockout mutants *FMO_GS-OX6_* and *FMO_GS-OX7_* were, respectively, analyzed but no significant changes in the GSL profiles were detected (Supplementary Tables S4 and S5). Considering that there are five other identified FMO_GS-OX_ enzymes with the same catalytic activity (Hansen et al., 2007; Li et al., 2008), the absence of observable phenotypic effects in the T-DNA knockout mutants could be attributable to the redundancy in gene function.

### Expression Pattern of *FMO_GS-OX6_* and *FMO_GS-OX7_*

**FIGURE 2:**
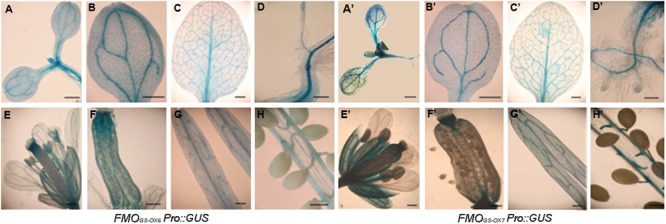
**Expression patterns of *FMO_GS-OX6_* and *FMO_GS-OX7_* detected by promoter-driven *GUS* expression. (A,B,A’,B’)** Cotyledons; **(C,C’)** foliage leaf; **(D,D’)** root and hypocotyl; **(E,E’)** flower; **(F,F’)** pistil; **(G,G’)** silique wall; **(H,H’)** Pseudoseptum and seeds; Bar = 0.3 mm.

To investigate the spatial expression patterns of *FMO_GS-OX6_* and *FMO_GS-OX7_*, we generated the transgenic plants *FMO_GS-OX6_Pro::GUS* and *FMO_GS-OX7_Pro::GUS*, respectively, expressing the *GUS* under the control of *FMO_GS-OX6_* and *FMO_GS-OX7_* promoters. Promoter activities were detected by *GUS* staining, a histochemical technique. As shown in **Figure 2**, the two genes presented similar expression patterns. In seedlings, the GUS signal was detected in the vascular tissue throughout the radicle, hypocotyls, cotyledon, foliage leaf, and root in both *FMO_GS-OX6_Pro::GUS* and *FMO_GS-OX7_Pro::GUS* transgenic plants (**Figures 2A–D,A’–D’**). This expression pattern partially overlaps with that of other *FMO_GS-OX_* (Li et al., 2011). Similarly, *FMO_GS-OX5_, FMO_GS-OX6_*, and *FMO_GS-OX7_* were expressed in the vascular tissue of the foliage, including the mid-vein and almost all side-veins, while the other four *FMO_GS-OX_* genes were expressed either only in the mid-vein and some major big veins or only in the side-veins (Li et al., 2011). Both the genes were expressed in the vascular tissue throughout the whole root (**Figures 2D,D’**), which is again similar to the expression pattern of *FMO_GS-OX5_* and different from that of the other *FMO_GS-OX_* genes, which are expressed only in the connections of the taproot and lateral roots (Li et al., 2011).

For both *FMO_GS-OX6_Pro::GUS* and *FMO_GS-OX7_Pro::GUS*, in flowers, *GUS* signals were detected in the vascular bundles of calyces, carpels, and stamen filaments, but very little signal was detected in petals (**Figures 2E–G,E’–G’**). Dense *GUS* signal was clearly observed at the top and bottom of the ovary and seed funicles (**Figures 2F,H,F’,H’**). This expression pattern is quite similar to that of other *FMO_GS-OX_* genes (Li et al., 2011). In general, the spatial expression of *FMO_GS-OX6_* and *FMO_GS-OX7_* overlapped with each other to a large extent and showed a similar pattern in the vascular tissue to that of other GSL biosynthetic genes (Mikkelsen et al., 2000; Reintanz et al., 2001; Tantikanjana et al., 2001; Grubb et al., 2004; Levy et al., 2005; Skirycz et al., 2006; Li et al., 2011).

**FIGURE 3:**
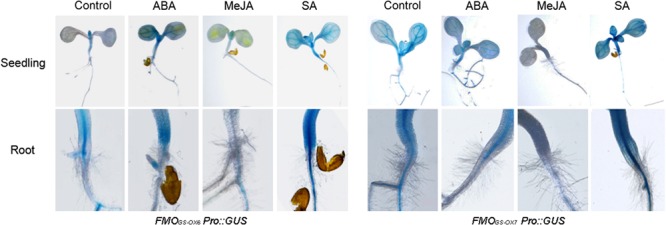
**Expression of *FMO_GS-OX6_* and *FMO_GS-OX7_* in response to exogenous abscisic acid (ABA), methyl jasmonate (MeJA), and salicylic acid (SA)**.

We further detected the expression of the two genes in response to exogenous ABA, MeJA, and SA. Both *FMO_GS-OX6_* and *FMO_GS-OX7_* were strongly inhibited by MeJA and induced by SA, whereas ABA promoted the expression of *FMO_GS-OX6_* significantly but did not affect *FMO_GS-OX7_* considerably (**Figure 3**).

### Responsive Expression of *FMO_GS-OX_* Genes to Different Stimuli

The *S*-oxygenation of MT GSLs to MS GSLs is highly important for plant defense because of the bioactivities of MS GSLs against both biotic and abiotic stresses (Stotz et al., 2011; Martínez-Ballesta et al., 2013, 2015). To date, including FMO_GS-OX6_ and FMO_GS-OX7_, seven FMO_GS-OX_ enzymes with redundant functions and overlapping spatial distribution have been identified. Therefore, we investigated whether the response of these seven *FMO_GS-OX_* genes to environmental stimuli was co-ordinated or varied. The promoter sequences of the seven *FMO_GS-OX_* genes were obtained from NCBI^2^ and analyzed using the web tool Plantcare ^3^. The elements in each of the promoter are listed in Supplementary Table S6.

**FIGURE 4:**
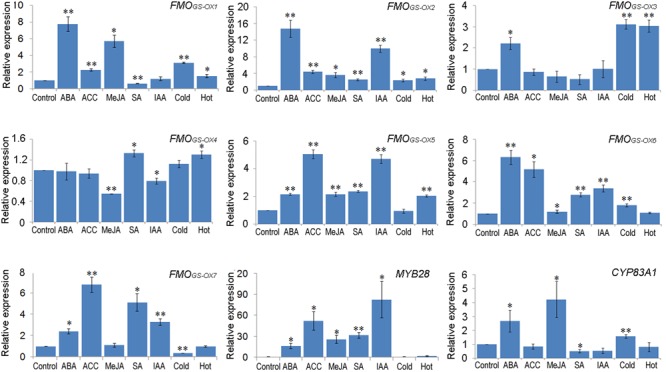
**Responsive expression patterns of *FMO_GS-OX_* genes to different exogenous stimuli**. The mRNA abundance of nine genes was analyzed by qRT-PCR. Samples for qRT-PCR were run in three biological replicates with three technical replicates and the data were represented as the Mean ± SD (*n* = 3). The relative gene expression was calculated using the ΔΔCt algorithm. The expression data were normalized using the invariant expression of *ACTIN II*. The leaves from mock control seedlings were used as reference sample, which was set to 1. Significant differences according to Student’s *t*-test are indicated: ^∗^*P* < 0.05, ^∗∗^*P* < 0.01.

Some common elements were found frequently among these promoters and the function of these elements is responsiveness to MeJA, ABA, auxin, ethylene, SA, cold, and heat stresses. To investigate the responsive expression of *FMO_GS-OX_* and other aliphatic GSL biosynthetic genes toward these hormone and environmental signals, 5-day old seedlings were treated with ABA, ACC, MeJA, SA, IAA, low temperature (4°C), and high temperature (30°C). Further, the expression level of the seven *FMO_GS-OX_* genes, together with that of *MYB28* (encodes the main transcription factor that regulates aliphatic GSL biosynthetic genes) and *CYP83A1* (an aliphatic GSL biosynthetic gene), were determined by qRT-PCR. Except *FMO_GS-OX4_*, which maintained relatively stable expression under different conditions, all the genes responded to the treatments (**Figure 4**). In general, the *FMO_GS-OX_* genes showed responsive expression pattern similar to that of the upstream genes (*MYB28* and *CYP83A1*) in the aliphatic GSL biosynthesis pathway. For example, almost all genes were upregulated by ABA and ACC. However, to the same stimulus, different genes presented different level of sensitivity. *FMO_GS-OX1_, FMO_GS-OX2_, FMO_GS-OX6_*, and *MYB28* showed upregulation (fivefolds), i.e., high sensitivity, toward ABA; *FMO_GS-OX5_, FMO_GS-OX6_, FMO_GS-OX7_*, and *MYB28*, toward ACC; *FMO_GS-OX1_* and *MYB28*, toward MeJA; *FMO_GS-OX7_* and *MYB28*, toward SA; and *FMO_GS-OX2_, FMO_GS-OX5_*, and *MYB28*, toward IAA. None of the aliphatic biosynthetic genes were very sensitive to cold and heat stresses.

## Discussion

Aliphatic GSLs display high structural diversity due to variation in chain length and secondary modification, and the bioactivity of these compounds is largely dependent on the structure of the side chain. The chemical modification catalyzed by the different FMO_GS-OX_ enzymes is highly important because MT GSLs and MS GSLs account for a large proportion of total GSLs, and production of MS GSLs is the basis of further modification of aliphatic GSLs.

The five annotated FMO_GS-OX_ enzymes were identified by Hansen (Hansen et al., 2007) and Li (Li et al., 2008). In the study conducted by Hansen, a phylogenetic tree of plant FMOs from rice, *Arabidopsis*, and poplar was analyzed. Within the clade considered to be involved in *S*-oxygenation, the presence of a subclade of seven proteins, including the annotated FMO_GS-OX1-5_, FMO_GS-OX6_, and FMO_GS-OX7_, only in the GSL-producing *Arabidopsis* plant suggests that these genes may be responsible for *S*-oxygenation of GSLs. Our result verified Hansen’s prediction. Compared with the known FMO_GS-OX_ enzymes, FMO_GS-OX6_ and FMO_GS-OX7_ was quite similar to FMO_GS-OX1-4_, which contribute to the conversion of both short and long-chain MT GSLs, and was different from FMO_GS-OX5_, which specifically recognizes long-chain substrates.

*FMO_GS-OX6_* and *FMO_GS-OX7_* showed very similar spatial expression patterns and it overlapped with that of most other GSL biosynthetic genes, which are frequently found in the vascular bundle. However, some small variations can be observed between these *FMO_GS-OX_* genes. Li et al. (2011) reported that in the leaf, *FMO_GS-OX1-4_* were distributed partially in the veins and *FMO_GS-OX5_* was expressed in the mid-vein and all the branched veins, whereas in the root, *FMO_GS-OX1-4_* was limited to the connections between the taproot and lateral roots and FMO*_GS-OX5_* was present throughout the vascular tissue in the whole root. Interestingly, FMO_GS-OX6_ and FMO_GS-OX7_, like FMO_GS-OX1-4_, catalyze *S*-oxygenation of GSLs without substrate specificity and exhibit less specific spatial expression like FMO_GS-OX5_. Based on the phylogenetic analysis of the *FMO_GS-OX_* genes in *Arabidopsis* (**Figure 1A**), our results suggest an FMO_GS-OX_ ancestor with a broad range of non-specific substrates for catalysis and broad spatial expression. The gene encoding this ancestor could probably have been duplicated into two groups: one (comprising *FMO_GS-OX1-5_*) might have become more specific either in spatial expression (*FMO_GS-OX1-4_*) or in substrate recognition (*FMO_GS-OX5_*) and the other group (comprising *FMO_GS-OX6_* and *FMO_GS-OX7_*) remained non-specific in both aspects.

Some common elements were detected in the promoter sequences of these *FMO_GS-OX_* genes. Consistently, the *FMO_GS-OX_* genes presented similar responsive expression tendency under several hormone treatments and temperature stresses. However, the sensitivity of each gene differed in response to the same treatment.

Integrating the knowledge of the various *FMO_GS-OX_* genes, we observed that they are quite redundant in their catalytic activity, distribution pattern, and response to exogenous stimuli. It is considered that duplicate genes play a more important role for the compensation of secondary metabolites than that of primary metabolites because there are fewer alternative pathways in secondary metabolite synthesis (Hanada et al., 2011). This redundancy of *FMO_GS-OX_* genes might play a significant role in functional compensation and increasing genetic robustness. Additionally, the variations found among these *FMO_GS-OX_* genes could possibly result in delicate functional difference and contribute to the finely tuned modification of aliphatic GSLs.

Aliphatic GSLs are primarily considered to contribute to plant resistance toward pests (Beekwilder et al., 2008). Recent studies discovered multiple functions of these compounds; they are involved in toxicity against pathogens and have been suggested to contribute to maintaining water balance under salt stress (Tierens et al., 2001; Stotz et al., 2011; Martínez-Ballesta et al., 2015). The versatile bioactivity of aliphatic GSLs is largely dependent on the length and modification of their side chain (Stotz et al., 2011). However, the contribution of specific metabolites to the biotic or abiotic stress defenses is still poorly understood. Identification of new FMO_GS-OX_ enzymes provides a way to investigate the specific bioactivity of aliphatic GSLs with different side chain structures. As an application for humans, these *FMO_GS-OX_* genes can potentially be used in breeding *Brassica* vegetables with improved anti-cancer properties conferred by the MS GSLs.

## Author Contributions

JL and WK carried out experiments and analyzed experiment results. QY, WC, and RX carried out partial of the experiments. JL and WK wrote the manuscript. WJ designed the experiments.

## Conflict of Interest Statement

The authors declare that the research was conducted in the absence of any commercial or financial relationships that could be construed as a potential conflict of interest.

## Footnotes

1 https://www.arabidopsis.org/

2 http://www.ncbi.nlm.nih.gov/

3 http://bioinformatics.psb.ugent.be/webtools/plantcare/html/

## Supplementary Material

FIGURE S1 **Expression levels of *FMO_GS-OX6_* and *FMO_GS-OX7_* in the overexpression mutants**.
