# Supplementary material for: Two Novel Flavin-Containing Monooxygenases Involved in Biosynthesis of Aliphatic Glucosinolates
Source: Front Plant Sci. 2016 Aug 29;7:1292. doi: 10.3389/fpls.2016.01292 (PMC5003058; doi:10.3389/fpls.2016.01292)
Supplement: Supplementary file 1 [file Table_1.DOCX]

Supplementary Table 1. Primers used in responsive expression analysis

| Gene name | Primer sequence |
| --- | --- |
| *FMO_GS-OX1_* | F：TCTGGAACTACTCATCTAAAGCTGACTCTG  R：ATGTCATGAATGCGTGGCACG |
| *FMO_GS-OX2_* | F：AGGCGGGTTACACTTCCCACAGA  R：AACGAGCGGACAACCACATTCTT |
| *FMO_GS-OX3_* | F：TTTGCCAGCAATGGGGATAGTAT  R：CTTCTATGAGTTCATCGTCGTCCC |
| *FMO_GS-OX4_* | F：CGTCAAGAGACTCTAGAAGGTATCCGA  R：ATTTGAGCGATGTTTGGTTCTGC |
| *FMO_GS-OX5_* | F：ACATAACGGGAGTGGCTAAAGAAG  R：ATTGCAGTGTCATCCAGGGTAAA |
| *FMO_GS-OX6_* | F：CATCTTTGAGCGTCAGAAACAAGTAGGA  R：CTCATGACTTCTCTGTGATCCGGGTAC |
| *FMO_GS-OX7_* | F：CAACCTACGGATTCACCCTACGA  R：AGCTACAGCCTCTTCCATCATCTTATC |
| *MYB28* | F：GCAGATTCGCAATGAAGAGGATAGT  R：GACTTCTTGGGAAACATCGGACATA |
| *CYP83A1* | F：ATACGGTCCAATCTTGTCATACAGGAT  R：TAATGCCATGTCACGCCTGC |

F: forward primer； R: reverse primer
