# Supplementary material for: Two Novel Flavin-Containing Monooxygenases Involved in Biosynthesis of Aliphatic Glucosinolates
Source: Front Plant Sci. 2016 Aug 29;7:1292. doi: 10.3389/fpls.2016.01292 (PMC5003058; doi:10.3389/fpls.2016.01292)
Supplement: Supplementary file 3 [file Table_3.DOCX]

**Supplementary Table S3.** Glucosinolates profile in the T-DNA mutant of *At1g12200*

| MT:(MS+MT) | Leaf Tissue | | |  | Seed Tissue | | |
| --- | --- | --- | --- | --- | --- | --- | --- |
|  | WT | mutant of *At1g12200* | *P*-value |  | WT | mutant of *At1g12200* | *P*-value |
| Propyl GSL (C3) | ND | ND |  |  | ND | ND | NS |
| Butyl GSL (C4) | 0.20±0.012 | 0.22±0.020 | NS |  | 0.68±0.034 | 0.60±0.028 | NS |
| Pentyl GSL (C5) | 0.36±0.022 | 0.35±0.014 | NS |  | 0.73±0.015 | 0.71±0.022 | NS |
| Hexyl GSL (C6) | ND | ND |  |  | ND | ND |  |
| Heptyl GSL (C7) | 0.29±0.034 | 0.27±0.040 | NS |  | 0.53±0.028 | 0.51±0.032 | NS |
| Octyl GSL (C8) | 0.11±0.013 | 0.12±0.008 | NS |  | 0.43±0.016 | 0.45±0.008 | NS |

Data presented are mean values of MT:(MS+MT) ± standard error for at least three replicates per sample. *P*-value for MT:(MS+MT) differences between the two genotypes were determined by Student’s t-test. ND means given GSL was not detectable; therefore, no statistical analyses were conducted. NS means non-significant *P*-value (*P*>0.05).
