# Supplementary material for: Two Novel Flavin-Containing Monooxygenases Involved in Biosynthesis of Aliphatic Glucosinolates
Source: Front Plant Sci. 2016 Aug 29;7:1292. doi: 10.3389/fpls.2016.01292 (PMC5003058; doi:10.3389/fpls.2016.01292)
Supplement: Supplementary file 4 [file Table_4.DOCX]

**Supplementary Table S4.** Glucosinolates profile in the T-DNA mutant of *FMO_GS-OX6_*

| MT:(MS+MT) | Leaf Tissue | | |  | Seed Tissue | | |
| --- | --- | --- | --- | --- | --- | --- | --- |
|  | WT | Mutant of  *FMO_GS-OX6_* | *P*-value |  | WT | Mutant of  *FMO_GS-OX6_* | *P*-value |
| Propyl GSL (C3) | ND | ND |  |  | ND | ND |  |
| Butyl GSL (C4) | 0.24±0.012 | 0.23±0.020 | NS |  | 0.73±0.044 | 0.74±0.036 | NS |
| Pentyl GSL (C5) | 0.22±0.012 | 0.20±0.022 | NS |  | 0.84±0.010 | 0.83±0.040 | NS |
| Hexyl GSL (C6) | ND | ND |  |  | ND | ND |  |
| Heptyl GSL (C7) | 0.26±0.040 | 0.28±0.034 | NS |  | 0.68±0.023 | 0.66±0.034 | NS |
| Octyl GSL (C8) | 0.14±0.013 | 0.15±0.010 | NS |  | 0.35±0.040 | 0.33±0.050 | NS |

Data presented are mean values of MT:(MS+MT) ± standard error for at least three replicates per sample. *P*-value for MT:(MS+MT) differences between the two genotypes were determined by Student’s t-test. ND means given GSL was not detectable; therefore, no statistical analyses were conducted. NS means non-significant *P*-value (*P*>0.05).
