# Supplementary material for: Two Novel Flavin-Containing Monooxygenases Involved in Biosynthesis of Aliphatic Glucosinolates
Source: Front Plant Sci. 2016 Aug 29;7:1292. doi: 10.3389/fpls.2016.01292 (PMC5003058; doi:10.3389/fpls.2016.01292)
Supplement: Supplementary file 6 [file Table_6.DOCX]

Supplementary Table S6. Promoter sequence analysis of *FMO_GS-OX_* genes

| **Gene Name** | **Motifs** | **Function** |
| --- | --- | --- |
| *FMO_GS-OX1_* | CGTCA-motif | MeJA-responsiveness |
|  | TGACG-motif | MeJA-responsiveness |
| *FMO_GS-OX2_* | ABRE | Abscisic acid responsiveness |
|  | AuxRR-core | Auxin responsiveness |
|  | CGTCA-mitif | MeJA-responsiveness |
|  | ERE | Ethylene-responsive |
|  | HSE | Heat stress responsiveness |
|  | LTR | Low-temperature responsiveness |
|  | TC-rich repeats | Defense and stress responsiveness |
|  | TCA-element | Salicylic acid responsiveness |
|  | TGA-element | Auxin-responsive element |
|  | TGACG-motif | MeJA-responsiveness |
| *FMO_GS-OX3_* | ABRE | Abscisic acid responsiveness |
|  | AuxRE | Part of an auxin-responsive element |
|  | AuxRR-core | Auxin responsiveness |
|  | G-box | Light responsiveness |
|  | HSE | Heat stress responsiveness |
|  | TC-rich repeats | Defense and stress responsiveness |
|  | TGA-element | Auxin-responsive element |
| *FMO_GS-OX4_* | ABRE | Abscisic acid responsiveness |
|  | Box-w1 | Fungal elicitor responsive element |
|  | CGTCA-motif | MeJA-responsiveness |
|  | ERE | Ethylene-responsive |
|  | G-box | Light responsiveness |
|  | GARE-motif | Gibberellin-responsive element |
|  | LTR | Low-temperature responsiveness |
|  | TCA-element | Salicylic acid responsiveness |
|  | TGACG-motif | MeJA-responsiveness |
| *FMO_GS-OX5_* | TC-rich repeats | Defense and stress responsiveness |
|  | TCA-element | Salicylic acid responsiveness |
| *FMO_GS-OX6_* | ABRE | Abscisic acid responsiveness |
|  | Box-w1 | Fungal elicitor responsive element |
|  | CGTCA-motif | MeJA-responsiveness |
|  | G-box | Light responsiveness |
|  | HSE | Heat stress responsiveness |
|  | TCA-element | Salicylic acid responsiveness |
|  | TGACG-motif | MeJA-responsiveness |
| *FMO_GS-OX7_* | GARE-motif | Gibberellin-responsive element |
|  | TC-rich repeats | Defense and stress responsiveness |
|  | TCA-element | Salicylic acid responsiveness |
