# Supplementary figures and images for: Two Novel Flavin-Containing Monooxygenases Involved in Biosynthesis of Aliphatic Glucosinolates
Source: Front Plant Sci. 2016 Aug 29;7:1292. doi: 10.3389/fpls.2016.01292 (PMC5003058; doi:10.3389/fpls.2016.01292)

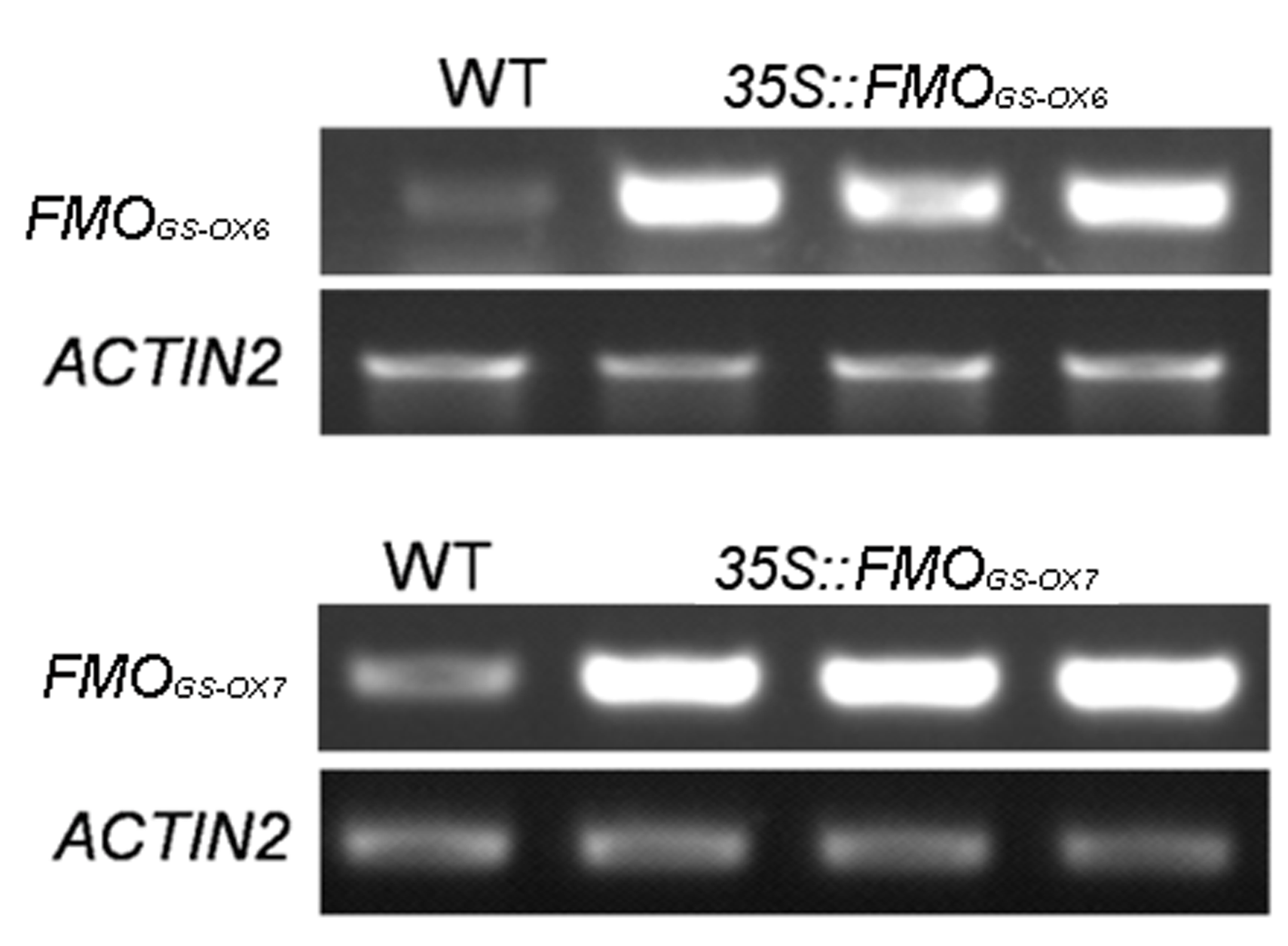

Supplement: FIGURE S1 — Expression levels of FMOGS-OX6 and FMOGS-OX7 in the overexpression mutants. [file Image_1.TIF]
